# Enhancement of the microwave photon-magnon coupling strength for a planar fabricated resonator

**DOI:** 10.1038/s41598-022-27285-6

**Published:** 2023-01-17

**Authors:** Aleksey Girich, Sergiy Nedukh, Sergey Polevoy, Kateryna Sova, Sergey Tarapov, Arthur Vakula

**Affiliations:** 1 O.Ya. Usikov Institute for Radiophysics and Electronics NAS of Ukraine, Kharkiv, 61085 Ukraine; 2 V.N. Karazin Kharkiv National University, Kharkiv, 61022 Ukraine; 3 Gebze Technical University, 41400 Gebze, Kocaeli Turkey

**Keywords:** Applied physics, Electrical and electronic engineering, Information storage

## Abstract

Planar resonators have a wide usage in modern microwave technologies and perspectives in novel quantum technologies development. As was demonstrated earlier, their utilization allows to achieve high values of microwave photon-magnon coupling strength—an important parameter in technologies of information coherent transfer from electromagnetic GHz range to the optical range. In the present work, the achievement of the high value of the microwave photon-magnon coupling strength by exploiting the increase of the spatial concentration of the magnetic component of the planar resonator electromagnetic field is reported. Starting from the conventional planar split-ring resonator design we increased the coupling strength up to 40% by modifying the resonator shape. The numerical simulation and experimental verification showed a predicted increase in the spatial concentration of the microwave magnetic component and showed the increased value of the microwave photon-magnon coupling strength as a sequence.

## Introduction

At present, great progress in quantum technologies has been achieved in the field of application of superconducting qubits that operate in the GHz range. However, the main disadvantage of this approach lies in the fact that superconducting qubits quantum state cannot be transmitted over long distances^1^ or stored for a long time. One of the methods to overcome this disadvantage is the creation of the information coherent transfer from electromagnetic GHz range to the optical. To solve this problem, as a rule, microwave (MW) resonators loaded by a ferromagnetic sample under ferromagnetic resonance (FMR) conditions are used. Thus, there is a coherent transfer of qubits information from the electromagnetic (EM) carrier to the magnetostatic spins oscillations in a ferromagnet, i.e. to the magnons. Subsequently, magnons modulate optical radiation.

In this method, one of the most important parameters is the quality of the MW to the IR radiation coherent conversion, which, in its turn, is also determined by the magnitude of the MW photon-magnon coupling strength.

To achieve a high value of the MW photon-magnon coupling strength, a so-called strong coupling regime^2^, which can be realized by using planar resonators^2,3^ with the ferrimagnetic (most often the yttrium iron garnet ferrite—YIG) element, is often used. Since the magnitude of the MW photon-magnon coupling strength is directly proportional to the filling factor of the ferrimagnet by the magnetic component *h* of the planar MW resonator EM field^4^, it is important to make the resonator EM field decrease rapidly with increased distance from the planar resonator surface for the case of micron-size thickness film-like ferrimagnet. Such a fast decay of the magnetic component *h* is observed for some planar resonators, in particular, for a split-ring resonator (SRR)^3^. Another advantage of planar resonators is their ease of manufacture that makes them suitable for use in integrated circuits.

The novelty of this work is the achievement of the high value of the MW photon-magnon coupling strength by exploiting the additional increase of the spatial concentration of the magnetic component of the planar resonator EM field. The aim of this paper is the development of the approach that enables to increase the MW photon-magnon coupling strength for a system of the SRR-YIG ferrite by modification of the SRR design.

## Results and discussion

To quantify and compare the degree of MW photon-magnon coupling, we will use the value *g*—the strength of photon-magnon coupling^2–5^. As shown earlier in^5^, the expression, which allows to extract the value of *g* from experimentally or numerically obtained transmission spectra (including for the case of interacted MW photon mode of SRR and magnon mode of the ferrimagnetic film) is:

1 $${{f}}_{{1}\left({2}\right)} =\frac{{{f}}_{1}^{0} + {{f}}_{2}^{0}}{2} \pm \sqrt{\frac{{{f}}_{1}^{0}-{{f}}_{2}^{0}}{2} + \Delta^{2}},$$

where ${f}_{1}$ and ${f}_{2}$ are the frequencies of the coupled resonances, ${f}_{1}^{0}$ and ${f}_{2}^{0}$ are the respective resonance frequencies in the absence of coupling, $\Delta$ is the coupling strength and can be rewritten as $\Delta =\frac{g}{2\pi }$. The value of *g* is defined in the frequency units and can be estimated by the fitting of the transmission spectra by the expression (1).

More detailed information on the procedure for obtaining the expression for the direct calculation of the magnitude of the photon-magnon coupling strength *g* is given in the Methods section (see later in the article).

The value of coupling strength *g* is directly proportional to the filling factor *η* of the ferrimagnet by the magnetic component of the SRR EM field that must be perpendicular to the external DC magnetic field (that should satisfy the FMR condition)^4^:

2 $$g = \frac{\gamma }{2}\eta \sqrt {\mu_{0} S\hbar \omega_{p} } ,$$

where *γ* is the gyromagnetic ratio, *S* is the value proportional to the magnetic moment of the ferrimagnet *μ* and the concentration of spins in it *n*_*s*_, $\hbar$ is the reduced Planck constant, *μ*_0_ is the vacuum permeability. From the dependency (2) it can be seen that a convenient way to increase the value of the MW photon-magnon coupling strength is to increase the filling factor of the ferrimagnet with the magnetic component of the SRR EM field that may be achieved by increasing the magnetic component of the SRR EM field spatial concentration.

So, using expression (1), one can extract the photon-magnon coupling strength from the obtained results (simulated and experimental), for example, the transmission spectrum at various values of the external magnetic field^6^.

Hence, the next step in our work is the modification of the SRR design in order to increase the magnetic component of the SRR EM field spatial concentration.

### Numerical and experimental results

To verify the abovementioned assumption, the numerical EM simulation was carried out first. The square-shaped SRR (SS-SRR)^3^ was chosen as the reference because it is one of the frequently used planar resonators in earlier papers. In order to increase the EM field spatial concentration for this type of resonators, numerical simulations were also carried out for its shape modification. In this case, the design of the resonator has been changed, while maintaining the resonance frequency of the resonator (about 6 GHz for our case). In both cases, the feeding microstrip line was 3.33 mm wide. For the SS-SRR the distance between the microstrip line and the SRR was 0.5 mm, and for the modified SRR (M-SRR) – 0.7 mm.

**Figure 1:**
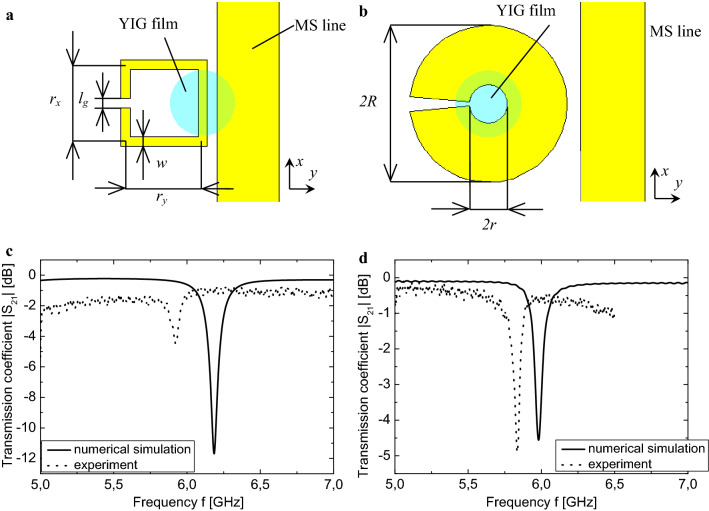
Top views of SRR: (**a**) SS-SRR; (**b**) M-SRR; (**c**) the transmission coefficient spectrum |*S*_21_| as a function of MW frequency *f* for SS-SRR (without the YIG); (**d**) the transmission coefficient spectrum |*S*_21_| as a function of MW frequency *f* for M-SRR (without the YIG).

In the beginning, we consider the case when there is no ferrimagnet, placed on the resonators. SS-SRR formed by the copper conductor with width *w*, bent into the shape of a square, with a gap width *l*_*g*_ (Fig. 1a). Rogers RO4350B with a thickness of 1.524 mm and 35 µm thick copper layer was used as a dielectric substrate material. The resonator parameters for the SS-SRR are: *r*_*x*_ × *r*_*y*_ = 4.1 × 4.1 mm^2^, the conductor line width is *w* = 0.5 mm, the gap width is *l*_*g*_ = 0.5 mm, and the resonance frequency is *f*_r_ = 6.2 GHz. The transmission coefficient (|*S*_21_| parameter) spectrum of the EM wave propagating through the feeding microstrip line as a function of MW frequency *f* for the SS-SRR resonator is shown in Fig. 1c.

**Figure 2:**
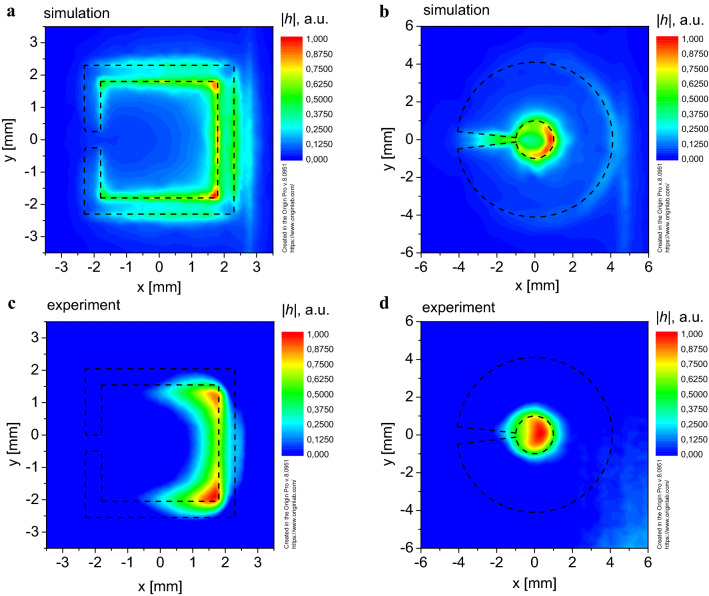
Spatial distribution of the magnetic component intensity *h* of the EM field in the plane of the SRR: (**a**) *h-*component, simulation for the SS-SRR; (**b**) *h-*component, simulation for the M-SRR; (**c**) *h-*component, experiment for the SS-SRR; (**d**) *h-*component, experiment for the M-SRR.

The spatial distribution of the *h*-component intensity of the EM field on the SS-SRR surface at the resonance frequency is shown in Fig. 2a. The magnetic component of the SS-SRR EM field is localized inside the SS-SRR with a maximum near the resonator side opposite to the gap.

To verify the increasing of the spatial concentration of the *h*-component of the EM field, the M-SRR (Fig. 1b) was numerically simulated as well. This resonator was shaped as a copper disk (radius *R* = 4.1 mm) with a circular hole (radius *r* = 1.0 mm) in the center and a radial cut (sector of 12°). The resonance frequency was adjusted to *f*_r_ = 6.0 GHz. The transmission coefficient (the |*S*_21_| parameter) spectrum of the EM wave propagating through the feeding microstrip line as a function of MW frequency *f* for the M-SRR resonator is shown in Fig. 1d.

The spatial distribution of the *h*-component intensity of the EM field in the resonator plane at the resonance frequency for the M-SRR is shown in Fig. 2b. It can be seen that the magnetic component of the M-SRR EM resonance field is mainly localized in the circular central hole. Thus, we can confirm the increase of the spatial concentration of the *h*-component of the EM field for M-SRR because the area of the *h*-component of the EM field localization for the M-SRR case is smaller than for the SS-SRR.

**Figure 3:**
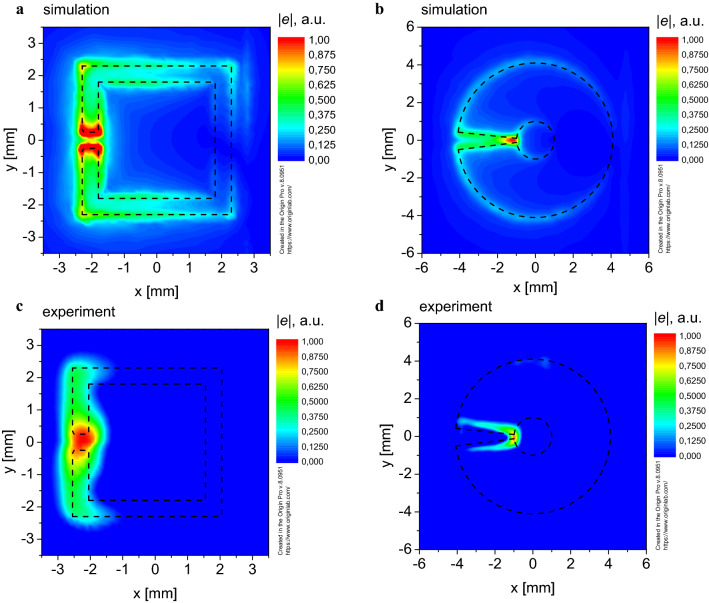
Spatial distribution of the electric component intensity *e* of the EM field in the plane of the SRR: (**a**) *e-*component, simulation for the SS-SRR; (**b**) *e-*component, simulation for the M-SRR; (**c**) *e-*component, experiment for SS-SRR; (**d**) *e-*component, experiment for the M-SRR.

Besides, we have calculated not only the distribution of the EM field magnetic component, but also the electric component spatial distribution. Figure 3a,b indicate that the electric *e*-component of the field is localized near the gaps for both resonators.

At the next step, for both SS-SRR and M-SRR, the ferrimagnetic sample shaped as the 0.01 mm thick YIG ferrite film disk of 3.5 mm in diameter was placed on the resonator surface in the area of the maximal localization of the magnetic component of the SRR EM field. For numerical simulation, we have selected the YIG parameters that are typical for an epitaxial grown single-crystal film. The direction of the external DC magnetic field *H* is chosen to be perpendicular to the microstrip line (along the *y*-axis), which corresponds to the standard FMR geometry with mutually perpendicular DC magnetic field and MW magnetic field (*h*_*z*_-component of the SRR EM field in our case).

**Figure 4:**
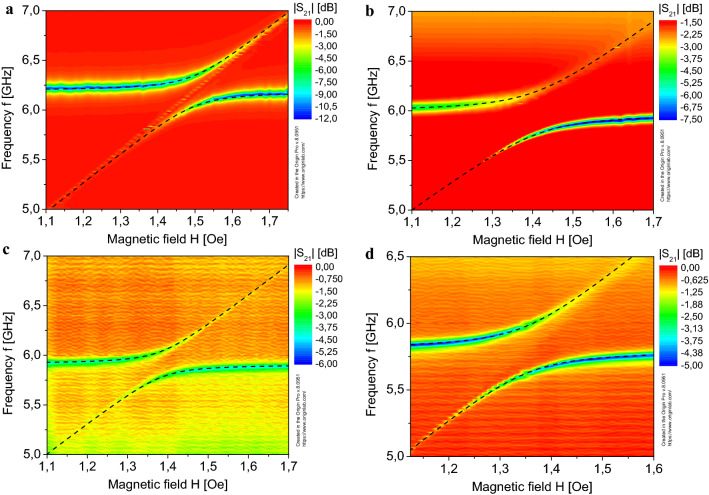
Transmission coefficient spectrum |*S*_21_| as a function of the external DC magnetic field *H* and the MW frequency *f* for SRR: (**a**) simulation for SS-SRR; (**b**) simulation for M-SRR; (**c**) experiment for SS-SRR; (**d**) experiment for M-SRR.

The simulated transmission coefficient (the |*S*_21_| parameter) spectrum of the EM wave propagating through the feeding microstrip line as a function of the DC magnetic field *H* and the MW frequency *f* are shown for the SS-SRR resonator in Fig. 4a, and for the M-SRR resonator in Fig. 4b. Because of the coupling of the SRR MW photon mode and the YIG magnon mode in the case of strong coupling the effect of modes repulsion occurs.

In Fig. 4a,b, the dotted lines show frequencies of the resonant modes according to (1). As is known, the value of the coupling strength *g* determines the value of the mode repulsion, and is given by the frequency gap Δ*f* = *2 g*/(2π), as shown in Fig. 4a,b^2,5^. The spin-number-normalized coupling strength was calculated by the formula $g_{N} /(2\pi ) = g/(2\pi \sqrt {N_{S} } )$^2^, where *N*_*S*_ is the number of spins in the ferrite sample volume, and the concentration of spins for the YIG in our simulation is *n*_*s*_ = 2.1 × 10^28^ m^−3^.

From the numerically calculated transmission spectra (Fig. 4a,b), we obtained the coupling strength *g* for resonators shown in Fig. 1a,b. For the SS-SRR (Fig. 1a), the coupling strength is *g*/(2π) = 155 MHz, and the spin-number-normalized coupling strength is *g*_*N*_/(2π) = 0.109 Hz. For the modified SRR (Fig. 1b), the coupling strength is *g*/(2π) = 216 MHz, and the spin-number-normalized coupling strength is *g*_*N*_/(2π) = 0.152 Hz.

The calculated data show that for the modified version of SRR the value of the MW photon-magnon coupling strength is 40% higher. This result can be explained by increasing of the filling factor of the YIG by the EM field *h*-component that is reached by a significant rise of the spatial concentration of the EM field *h*-component for the M-SRR (Fig. 2b).

**Figure 5:**
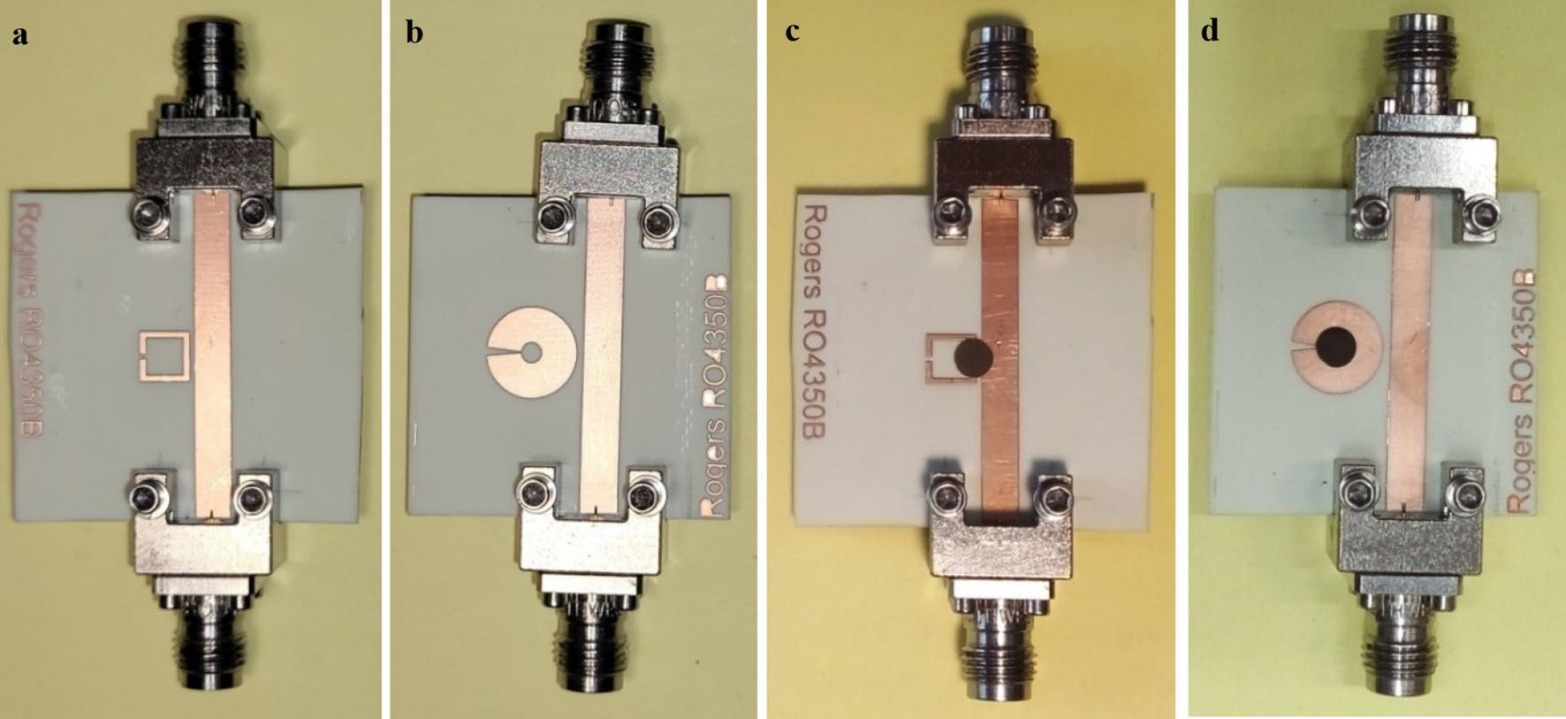
Photo of the planar resonators: the SS-SRR (**a**), the M-SRR (**b**), the SS-SRR with YIG sample (**c**) and the M-SRR with YIG sample (**d**).

To verify the results of the numerical calculation, the experiment has been carried out. Both resonators were fabricated on the Rogers RO4350B dielectric substrate together with the microstrip line that feeds the resonator. The P9374A Keysight VNA was connected to both ends of the feeding microstrip line through the coaxial-to-microstrip adapters (Fig. 5).

The experimental transmission spectra of the SS-SRR and M-SRR without YIG film are shown in Fig. 1c,d, respectively. These spectra are in good agreement with the spectra obtained by numerical simulation (Fig. 1c,d).

In the experiment, we used the 10 μm thick epitaxial grown single-crystal YIG film deposited on the gallium gadolinium garnet (GGG) disk substrate of 3.5 mm in diameter. The experimentally measured FMR linewidth for the YIG film sample is about 3 MHz. It is sufficient to achieve the strong coupling regime between resonant modes, since the damping rate (the linewidth measured at the half amplitude level) of the resonator (Fig. 1c,d) and the FMR linewidth (also measured at the half amplitude level) are less than the coupling strength *g*/2π^7,8^. The sample was placed on top of the resonators in the area with the highest density of the *h*-component of the SRR EM field, as it is shown in Figs. 1a,b and Fig. 2. The resulting transmission spectra (Fig. 4c,d) show the coupling strength g/2π = 122 MHz for the SS-SRR and g/2π = 183 MHz for the M-SRR. The spin-number-normalized coupling strength is *g*_*N*_ = 0.086 Hz for the SS-SRR and *g*_*N*_ = 0.129 Hz for the M-SRR. This differs by 27% downward from the numerical simulation for the SS-SRR.

**Figure 6:**
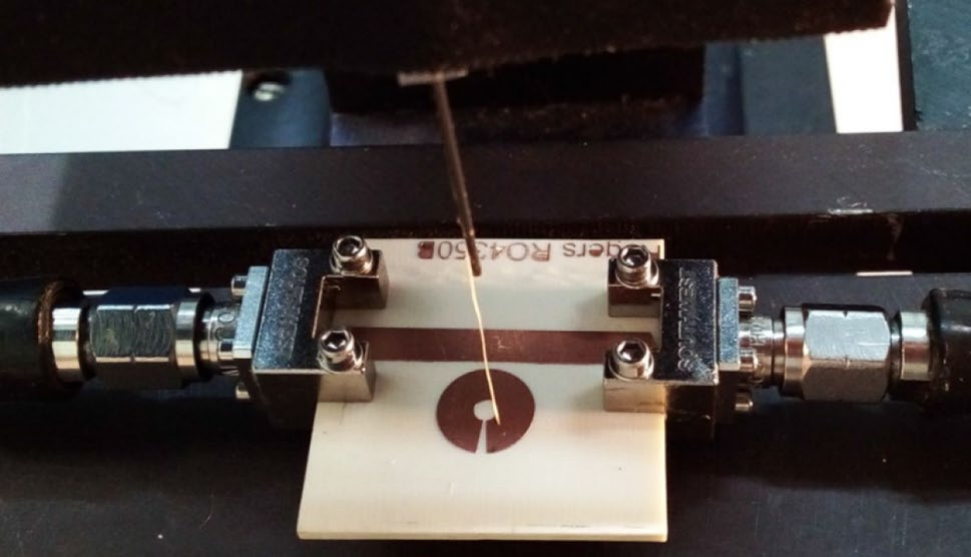
Photo of the M-SRR with the probe.

We used the passive probe technique to study the spatial distribution of the EM field components above the resonators plane^9,10^. The spatial distribution of the EM field was obtained using the specially designed position adjustment stage system^10^. The photo of M-SRR with the probe is shown in Fig. 6.

For this purpose, two probes (made of 1SCh4 brand ferrite and made of graphite) small (~ 0.2 mm) in comparison with the EM wavelength were used in the position adjustment stage. It is well known that the ferrite in the absence of the DC external magnetic field possesses significant magnetic losses, while the graphite possesses significant electrical losses. Therefore, the probe made of ferrite was used to obtain experimentally the spatial distribution of the *h*-component of the EM field (Fig. 2c,d), and the probe made of graphite was used to experimentally obtain the spatial distribution of the EM field’s *e*-component (Fig. 3c,d).

Figure 2c demonstrates that the *h*-field spatial concentration is observed on the opposite side of the gap of the SS-SRR. For the M-SRR, the *h*-field concentration is observed in its center (Fig. 2d). Both of these results were previously predicted by numerical calculations (compare Fig. 2a and c, Fig. 2b and d). So, for achieving the highest value of the MW photon-magnon coupling *g* we have to place the YIG film sample in this area to reach the most intense interaction of the M-SRR *h*-field with magnons in the ferrimagnet.

Figure 3c,d indicate that the *e*-component of the field is concentrated near the gaps for both the SS-SRR and the M-SRR, which is in good agreement with the results of the numerical simulation (Fig. 3a,b).

## Conclusions

Thus, high values of strong MW photon-magnon coupling strength *g* were predicted numerically and achieved experimentally using the modified split-ring resonator with a ferrimagnet sample.

It was shown that the modification of the split-ring resonator design enables to achieve high spatial localization of the *h*-component of the EM field and to increase the filling factor of the ferrimagnet by the resonant SRR EM field.

In this case, numerical and experimental studies showed that the magnitude of the MW photon-magnon coupling strength *g* in a such system increases up to 40% in comparison with the original SRR resonator, while maintaining its resonance frequency.

## Methods

### Analytical model of the MW photon-magnon coupling

There are several often-used analytical models describing the MW photon-magnon coupling^2^: based on the classical coupled harmonic oscillators theory, the dynamic phase correlation, the quantum based theory.

To describe the transmission spectra of the interacting system of the SRR and the YIG film, the model based on motion equations for coupled classical oscillators was first used in^5^. The motion equations in this case can be written in the following form, where one of the oscillators is acted on by an external force *F*_1_/*m*_1_:

3 $$\begin{aligned} & \ddot{x}_{1} (t) + \beta_{1} \dot{x}_{1} (t) - Kx_{2} (t) = F_{1} /m_{1} , \hfill \\ & \ddot{x}_{2} (t) + \beta_{2} \dot{x}_{2} (t) - Kx_{1} (t) = 0, \hfill \\ \end{aligned}$$

where *x*_1_(*t*) and *x*_2_(*t*)—are displacements of oscillators, *β*_1_ and *β*_2_ are the value of dissipation for oscillators, and *K* is the coupling value between them. However, this model does not take into account that an external force can act on both oscillators simultaneously.

If we take into account that the exciting force (the MW current in the feeding microstrip line and magnetic field induced by it) is acting on both oscillators simultaneously—SRR and YIG ferrite under the FMR condition, the model of coupled resonators under harmonic excitation by the EM field with a frequency *ω* is described by the following expression^6^:

4 $$\left( {\begin{array}{ll} { - \omega^{2} + i\beta \omega + \omega_{p}^{2} } & \quad {iK} \\ { - iK} & \quad { - \omega^{2} + i\alpha \omega + \omega_{r}^{2} } \\ \end{array} } \right)\left( {\begin{array}{*{20}c} {j_{R} } \\ {m_{m} } \\ \end{array} } \right) = \left( {\begin{array}{*{20}c} 1 \\ \tau \\ \end{array} } \right),$$

where *j*_*R*_—is the SRR current, *m*_*m*_—is the dynamically changing magnetization of the magnetic spins of the YIG under the FMR condition, *ω*_*p*_ is the angular resonance frequency of the SRR, *ω*_*r*_ is the FMR angular frequency, *α* is the loss factor of the magnons system, *β* is the loss factor in the SRR, *τ* is the coefficient for action force on the magnons system relative to the unit action force on the SRR resonator. Therefore, solution of (4) allows to consider more precisely both the spatial orientation of the SRR gap and the position of YIG film according to both the feeding line and the SRR.

In this paper, we used expressions derived from the classical coupled harmonic oscillators theory and presented in^5^, because the accuracy of the estimated coupling strength *g* was satisfactory for our research.

### Numerical calculations

Electrodynamical simulation of patterns of the electromagnetic field spatial distribution, as well as the calculation of the transmission spectra of electromagnetic waves propagating through the resonator with a magnetic sample in the presence of the external DC magnetic field, has been carried out numerically using the software package CST Studio Suite 2018 (the built-in frequency domain solver was used).

### Measurement setup

The measurements were performed at room temperature. During measurements the investigated planar device was mounted between the two poles of the electromagnet. The external static magnetic field is tuned by varying the electric current and the specially designed Gauss meter (based on a Hall probe) is used to monitor the applied external magnetic field. By varying the magnitude of the external static magnetic field, the FMR frequency was tuned to approach the resonance frequency of the planar resonator. Then the S_21_ parameter was recorded by sweeping the microwave signal frequency by the VNA.

## Data Availability

The datasets generated and/or analyzed during the current study are available from the corresponding author on reasonable request.
